# Natural fermentation quality, bacteria, and functional profiles of three cuttings of alfalfa silage in a year in Inner Mongolia, China

**DOI:** 10.3389/fmicb.2023.1083620

**Published:** 2023-03-09

**Authors:** Juanjuan Sun, Jing Wang, Chunsheng Bai, Jinmei Zhao, Ying Yun, Zhu Yu, Yanlin Xue, Tengwei Zhang, Wenlong Bao

**Affiliations:** ^1^Institute of Grassland Research of Chinese Academy of Agricultural Sciences, Hohhot, China; ^2^Inner Mongolia Academy of Grassland Science, Hohhot, China; ^3^College of Grassland, Resources and Environment,Inner Mongolia Agricultural University, Hohhot, China; ^4^College of Horticulture, Shenyang Agricultural University, Shenyang, China; ^5^College of Grassland Science and Technology, China Agricultural University China, Beijing, China; ^6^Inner Mongolia Academy of Agricultural and Animal Husbandry Sciences, Hohhot, China

**Keywords:** *Medicago sativa* L., bacterial community, cutting time, functional profile, natural fermentation quality

## Abstract

Alfalfa is harvested two or three times a year in central and western Inner Mongolia, China. However, the variations in bacterial communities as affected by wilting and ensiling, and the ensiling characteristics of alfalfa among the different cuttings, are not fully understood. To enable a more complete evaluation, alfalfa was harvested three times a year. At each time of cutting, alfalfa was harvested at early bloom, wilted for 6 h, and then ensiled in polyethylene bags for 60 days. The bacterial communities and nutritional components of fresh alfalfa(F), wilted alfalfa(W) and ensiled alfalfa(S), and the fermentation quality and functional profile of bacterial communities of the three cuttings alfalfa silage, were then analyzed. Functional characteristics of silage bacterial communities were evaluated according to the Kyoto Encyclopedia of Genes and Genomes. The results showed that all nutritional components, fermentation quality, bacterial communities, carbohydrate, amino acid metabolism and key enzymes of bacterial communities were influenced by cutting time. The species richness of F increased from the first cutting to the third cutting; it was not changed by wilting, but was decreased by ensiling. At phylum level, *Proteobacteria* were more predominant than other bacteria, followed by *Firmicutes* (0.063–21.39%) in F and W in the first and second cuttings. *Firmicutes* (96.66–99.79%) were more predominant than other bacteria, followed by *Proteobacteria* (0.13–3.19%) in S in the first and second cuttings. *Proteobacteria*, however, predominated over all other bacteria in F, W, or S in the third cutting. The third-cutting silage showed the highest levels of dry matter, pH and butyric acid (*p* < 0.05). Higher levels of pH and butyric acid were positively correlated with the most predominant genus in silage, and with *Rosenbergiella* and *Pantoea*. The third-cutting silage had the lowest fermentation quality as *Proteobacteria* were more predominant. This suggested that, compared with the first and second cutting, the third cutting is more likely to result in poorly preserved silage in the region studied.

## Introduction

Alfalfa (*Medicago sativa* L.) is a major legume forage source in the diets of ruminants in Inner Mongolia, China (Tao et al., 2018), owing to its high protein content, nutritional value, digestibility, and productivity (Agarussi et al., 2019a). In the central and western parts of Inner Mongolia it can be harvested two or three times a year. The weather conditions in this region sometimes do not permit three cuttings for hay production, so alfalfa may be ensiled. High moisture content is an unfavorable factor influencing alfalfa silage quality (Wang et al., 2009). It has been suggested that fresh alfalfa (F) needs to be wilted until dry matter (DM) values are higher than 300 g/kg fresh matter (FM) to inhibit the growth of some undesirable microorganisms (Luchini et al., 1997) and to prevent effluent production (Duniere et al., 2013). Alfalfa silage may experience *Clostridia* fermentation when the moisture content of the alfalfa plant exceeds 700 g/kg FM (Yang et al., 2020). During the wilting period the plant material is exposed to atmospheric oxygen and the plant cells continue to respire. The enzymes produced by plant respiration can break down proteins, sugars and hemicellulose, decreasing the nutritional value of the silage (Pitt et al., 1985; Mcgarvey et al., 2013).

High-throughput sequencing has been widely used in recent years to illustrate the microbial community structure in the alfalfa ensiling process (Guo et al., 2018; Yang et al., 2020, 2022), and research in this area has primarily focused on the microbial communities in alfalfa silage. Microbial communities are influenced by many factors such as additives (Yang et al., 2020), moisture content (Yang et al., 2022), and silage density (Sun et al., 2021). The dominant genera changed with the different inoculated lactic acid bacteria (LAB) additives (Ogunade et al., 2018; Fan et al., 2021). Only limited research has focused on the microbial communities of wilted alfalfa (W). Previous studies have indicated that wilting may affect the abundance of epiphytic microbes (Agarussi et al., 2019a), and that wilting inhibited the growth of spoilage microorganisms in the early stage of ensiling, which resulting in good fermentation quality (Yang et al., 2022).

Epiphytic microflora, the microorganisms naturally existing on forage crops, play an important role in silage fermentation quality and also influence the effectiveness of bacterial inoculation (Lin et al., 1992). Wilting affects the epiphytic microbial structure (Agarussi et al., 2019a), so epiphytic microbes of wilting plant will be the microorganisms that initiate the silage fermentation process. Changes in microbial communities of alfalfa before and after silage, and silage inoculated with LAB, have been studied in recent years (Stevenson et al., 2006; Bao et al., 2016). Bacterial populations vary according to geographic area, climate or growing stage (Duniere et al., 2013). Previous studies have reported that wilting of fresh alfalfa before ensiling may improve the fermentation quality of the silage (Agarussi et al., 2019a; Yang et al., 2022). All of these studies, however, used only one cutting of alfalfa, for example the first cutting (Li F. et al., 2020; Wang B. et al., 2020), the second cutting (Wang et al., 2021) or the fourth cutting (Li R. et al., 2020). Many research publications did not mention which alfalfa cutting was used in the experiment (Yang et al., 2019, 2020; Wang et al., 2022). We hypothesized that the bacterial communities differed among the annual three cuttings because the climate conditions were different. The effects of wilting on the bacterial community of W, however, remain unclear.

Recently, the studies have focused not only on changes in bacterial community, but also on bacterial interaction and functional prediction on alfalfa (Bai et al., 2021; Wang et al., 2022).The objective of this study was to compare the changes in bacterial communities from F, W, and S alfalfa among three cuttings in 1 year, and to determine the predicted functional profiles and fermentation characteristics of ensiled alfalfa. The results may provide a theoretical basis for techniques regulating the production of quality alfalfa silage from different cuttings, and also improve our understanding of bacterial communities in natural fermentation conditions with no additives to the silage. Our hypothesis was that the cutting time would influence the microbial communities, and that the difference in fermentation quality would be associated with differences in the bacterial communities.

## Materials and methods

### Material and silage preparation

A third-year stand of alfalfa cultivar *Medicago sativa* L. cv. “Zhongcao NO.3” was examined at the 10% bloom stage of maturity in the first, second and third cuttings from 2 June 2017 to 1 September 2017. The plants were grown on the experimental base of the Institute of Grassland Research of the Chinese Academy of Agricultural Sciences in Hohhot district (111°45′ E, 40°36′ N) on the Tumochuan plain, China. F was harvested 8 cm above ground level. It was mowed and swathed at 10:00 a.m. and then wilted in the windrow for 6 h prior to precision chopping. W was chopped into 1- to 2-cm lengths and mixed well, then packed into polyethylene bags and vacuum sealed. The samples were stored at room temperature for 60 days.

### Chemical composition, fermentation quality, and microbial enumeration analyses

F, W, and S specimens were dried at 65°C for 48 h and then weighed to determine DM content. The dried samples were then ground and passed through a 1-mm screen using a laboratory knife mill (QE-500, Yili Instruments, Zhejiang, China) for later analysis. Neutral detergent fiber (NDF) (Van Soest et al., 1991) and acid detergent fiber (ADF) (Robertson and Van Soest, 1981) were measured using an ANKOM fiber analyzer (ANKOM2000; Macedon, NY, United States). Crude ash (ash) content was determined by burning samples in a muffle furnace at 500°C for 5 h and then weighing the residue. Total nitrogen (TN) content was determined by the Kjeldahl procedure (Krishnamoorthy et al., 1982) and crude protein (CP) was determined by multiplying the total N by 6.25.

The fermentation quality of the silage was determined using distilled water extracts. We placed 20-g samples of S in separate containers of 180 mL double distilled sterile water and stored them in a refrigerator at 4°C for 24 h before filtering them through four layers of cheesecloth. After the sample extracts had been prepared they were used immediately for pH analysis, then passed through a 0.22-μm filter and stored at −20°C for organic acid and NH_3_-N analysis. The pH was measured with a glass electrode pH meter. The concentrations of lactic acid (LA), acetic acid (AA), propionic acid (PA) and butyric acid (BA) were analyzed with a high-performance liquid chromatography (HPLC) system with a Shodex Rspark KC-811 column (Showa Denko K.K., Kawasaki, Japan), eluted using 3 mmol/l HClO_4_ at flow rate of 1 mL/min at 50°C and detected at 210 nm using Waters E2695 (Waters Co. Ltd., Milford, United States).

Wet sample (20 g) of F, W and S was diluted using 180 mL sterilized distilled water and serially diluted from 10^−1^ to 10^−6^ in the sterilized water before microbial enumeration. The populations of LAB, coliform bacteria (CB), yeast and aerobic bacteria (AB) using the plate count method (Cai et al., 1999). The following culture media (Guangzhou Huankai Microbial Science and Technology Co. Ltd., Guangzhou, China) were used to isolate various microorganisms: De Man Rogosa Sharpe agar, violet-red bile agar, potato dextrose agar and nutrient agar for LAB, CB, yeast and AB, respectively. The plates for enumerating LAB were placed in an anaerobic box (C-31, Mistubishi Gas Chemical Co. Inc., Tokyo, Japan) and incubated at 37°C for 48 h in a general incubator. The plates for CB and AB were incubated at 37°C for 48 h in aerobic conditions in the same general incubator. The plate for yeasts were cultivated at 32°C for 48 h in aerobic conditions in another general incubator. Microorganism population numbers were expressed as colony forming units (cfu) per gram of FM (Xu et al., 2019).

### Microbial community analysis

The specimens of F, W, and S were stored at −80°C immediately and were used for the molecular analysis of the microbiota. The bacterial total genomic DNA was extracted using the E.Z.N.A.®Stool DNA Kit (D4015, Omega, Inc., United States) according to manufacturer’s guidelines.

The V3–V4 region of the prokaryotic (bacterial and archaeal) small subunit (16S)rRNA gene was amplified with primers 341F (5′-CCTACGGGNGGCWGCAG-3′) and 805R (5′-GACTACHVGGGTATCTAATCC-3′) (Logue et al., 2016). PCR amplification was performed in a total volume of 25 μL reaction mixture containing 25 ng of template DNA, 12.5 μL PCR Premix, 2.5 μL of each primer and PCR-grade water to adjust the volume. The PCR conditions to amplify the prokaryotic 16S fragments consisted of an initial denaturation at 98°C for 30 s; 32 cycles of denaturation at 98°C for 10 s, annealing at 54°C for 30 s and extension at 72°C for 45 s; and then final extension at 72°C for 10 min. The PCR products were confirmed with 2% agarose gel electrophoresis. The PCR products were purified by AMPure XT beads (Beckman Coulter Genomics, Danvers, MA, United States) and quantified by Qubit (Invitrogen, USA). The amplicon pools were prepared for sequencing and the size and quantity of the amplicon library were assessed in an Agilent 2100 Bioanalyzer (Agilent, USA) and with the Library Quantification Kit for Illumina (Kapa Biosciences, Woburn, MA, USA), respectively. The libraries were sequenced on the NovaSeq PE250 platform (Sun et al., 2022). Microbial functions were proof checked from the Kyoto Encyclopedia of Genes and Genomes (KEGG) database using Phylogenetic Investigation of Communities by Reconstruction of Unobserved States (PICRUSt)(Bai et al., 2021).The sequence data reported in this study were deposited in the NCBI Sequence Read Archive database (accession number: PRJNA827667).

### Statistical analyses

The data on the fermentation quality and microbial counts were analyzed as a 3 × 3 factorial design. The model comprised three cuttings, three sampling stages (include F, W, and S) and their interaction. The differences among the three cutting and three sampling stages were analyzed with the Generalized Linear Model(GLM) procedure of SAS (SAS System for Windows, version 8.0; SAS Institute Inc., Cary, NC, United States). The interaction between cutting and sampling stage was analyzed using the Pairwise Difference(PDIFF) procedure of SAS. Correlation analysis of the top ten genera, with fermentation quality and DM, was analyzed using R version 3.6.3.

## Results

### Sequencing results and bacterial diversity

**Figure 1:**
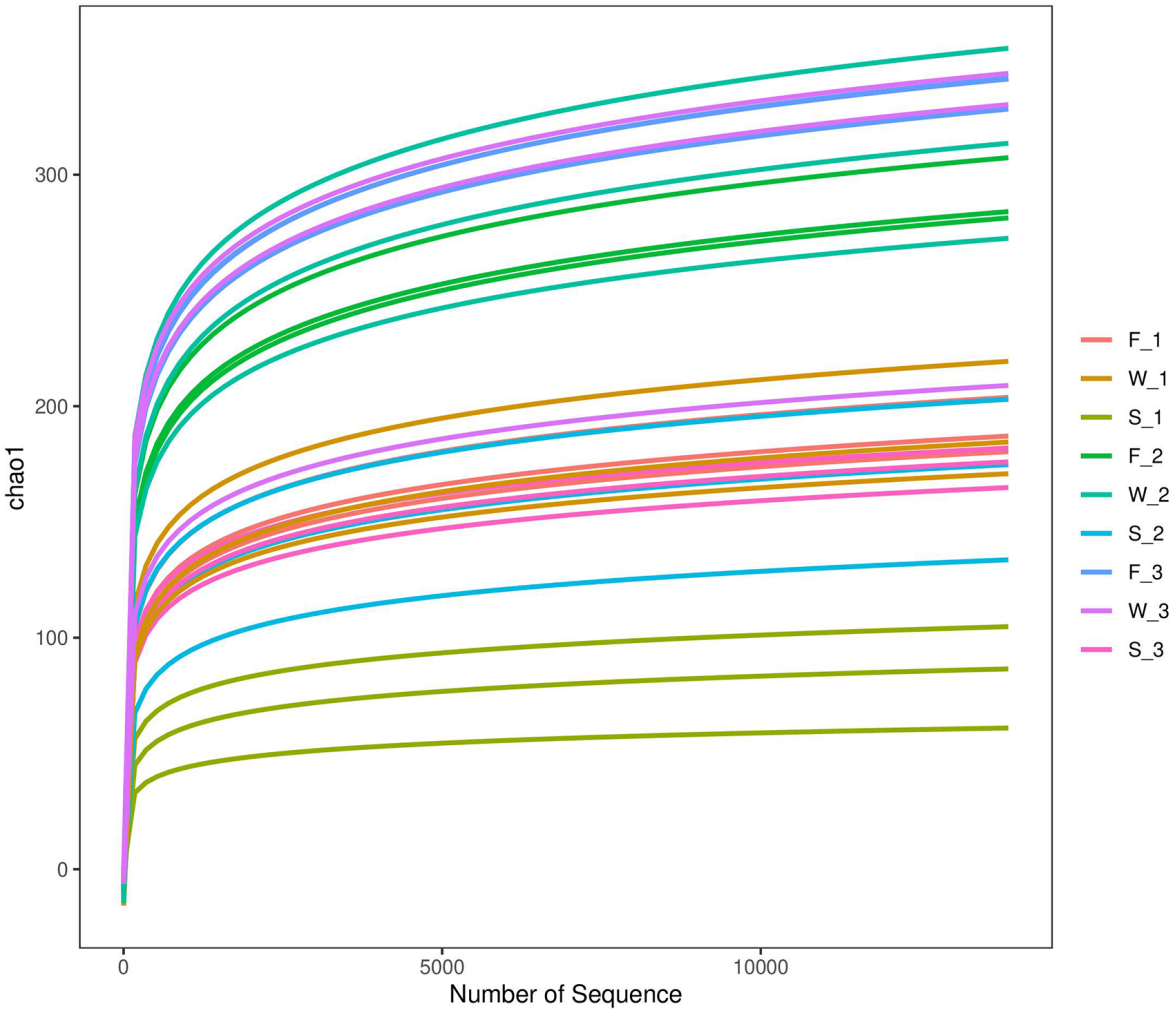
Chao1 rarefaction curve of samples for pre-ensiled crop fresh alfalfa (F), wilted alfalfa (W) and ensiled alfalfa (S) at the first cutting (F_1, W_1, and S_1), the second cutting (F_2, W_2, and S_2), or the third cutting (F_3, W_3, and S_3).

**Figure 2:**
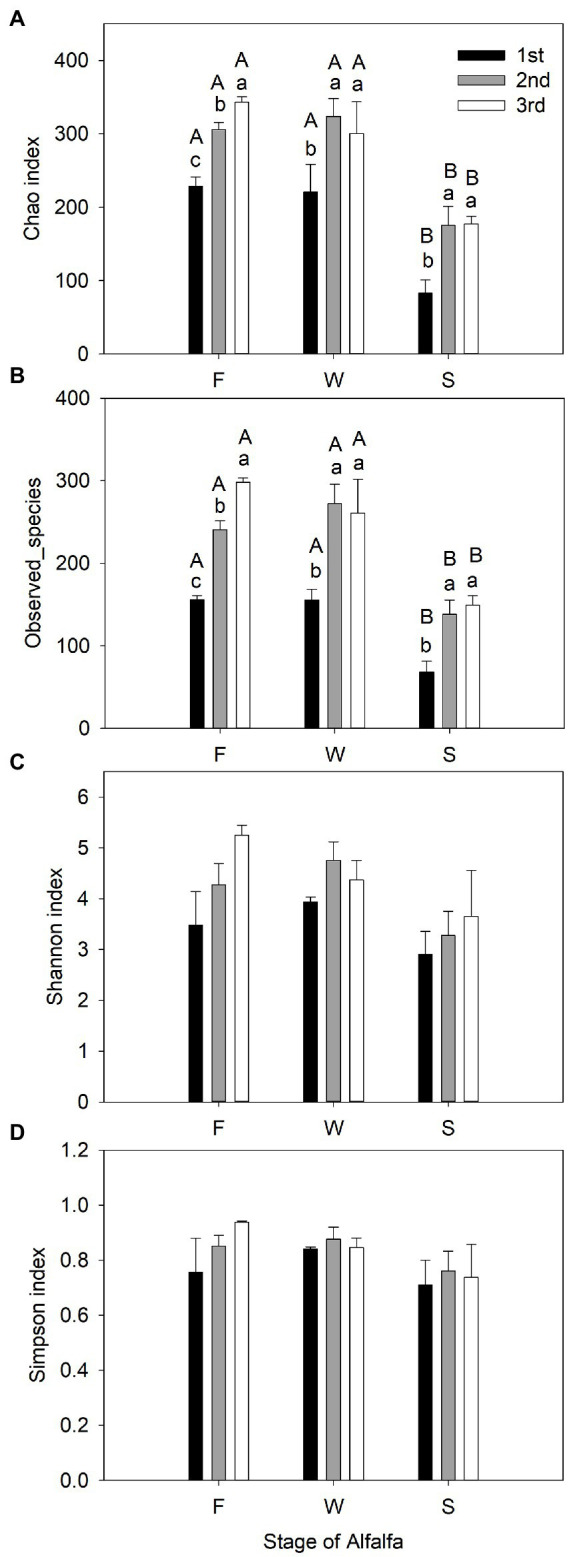
Observed bacterial community richness and diversity indexes (mean ± SE, *n* = 3) of fresh alfalfa (F), wilted alfalfa (W), and ensiled alfalfa (S). **(A)** Estimate of Richness Chao1 index. **(B)** Estimate of Richness Observed_species. **(C)** Estimate of diversity Shannon index. **(D)** Estimate of diversity Simpson index. The black filled bar indicate the first cutting alfalfa, the gray filled bar indicted the second cutting alfalfa, and the non-filled bar indicate the third cutting alfalfa. Bar with different capital letters differ among F, W, and S in the same cutting; Bar with different lowercase letters differ among different cuttings.

**Figure 3:**
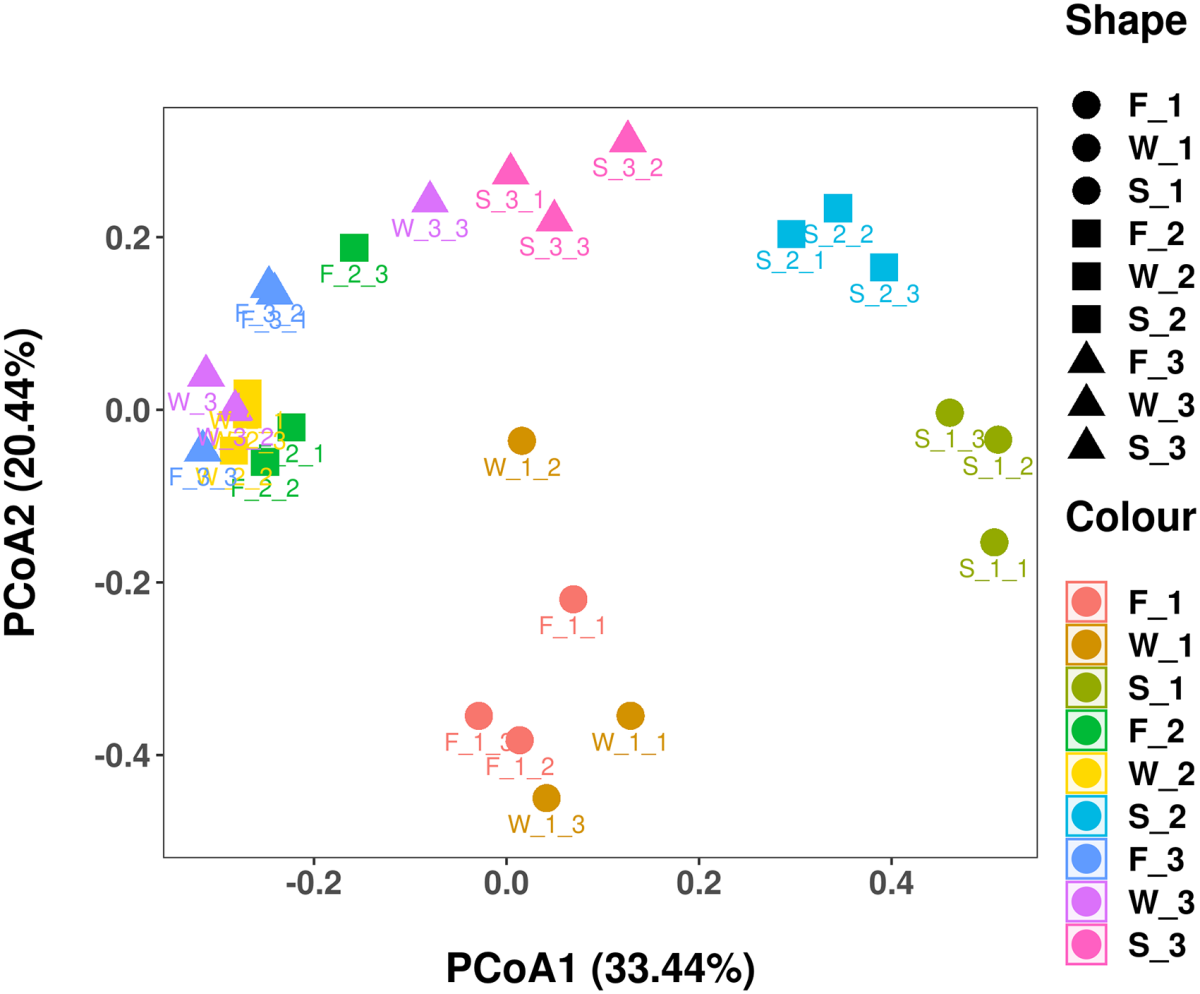
Unweighted unifrac principal coordinate analysis (PCoA) plot of individual samples in fresh alfalfa (F), wilted alfalfa (W), and ensiled alfalfa (S) at three cuttings. The PCoA plot indicates the phylogenetic distance (variation) between samples using 2 principal coordinates (PC1 and PC2). The percentage variation explained by each PC is indicated on each axis.

Sequencing of the V3–V4 region of the bacterial 16S rRNA gene of the 27 samples yielded 905,918 reads with an average of 33,553 ± 1,651 reads per sample after quality filtering. Rarefaction curves plateaued in all samples sequenced (shown as Chao1 index in Figure 1), indicating that the number of reads was sufficient for identifying operational taxonomic units (OTU). The average Good’s coverage for all samples was greater than 99%, indicating that the depth of sequencing was adequate for reliable analysis of the bacterial community (Supplementary Table S1). Figure 2 shows the alpha diversity of F, W and S bacterial communities. Compared with F and W, analysis revealed that S had lower (*p* < 0.05), Chao1 index and observed_species (Figures 2A,B); these measure the richness of the bacterial communities obtained for clustering at 97% similarity level. The Shannon index and Simpson index were unaffected by cuttings and by wilting and ensiling (Figures 2C,D). The unweighted PCoA UniFrace plot revealed compositional differences in the bacterial community of the 27 samples (Figure 3). The F and W samples appeared to cluster apart from S, and the F and W samples from the first cutting appeared to cluster apart from that of second and third cuttings.

### Comparisons at the phylum, the class, and the family level

The bacterial community structure of F, W, and S were represented by five phyla (Table 1). Proteobacteria (78.2–99.7%) were predominant, followed by Firmicutes (0.063–21.39%) in F and W in the first and second cuttings. Firmicutes (96.66–99.79%) were predominant in S in the first and second cuttings, followed by Proteobacteria (0.13–3.19%). However, Proteobacteria were predominant in F, W and S in the third cutting. There were decreased Proteobacteria in alfalfa silage in both first and second cuttings, but not in the third cutting. Both Proteobacteria and Firmicutes were interactionally affected (*p* < 0.05) by cutting and sampling stages. The relative abundances of Actinobacteria, Cyanobacteria and Bacteroidetes were similar for all cutting and sampling stages (*p* > 0.10).

**Table 1:** Relative abundance (%) of the bacterial community at phylum level of fresh, Wilted and ensiled alfalfa at three cuttings.

**Items**	**Cutting**	**Stages**			**SEM**	***p*-Value**	**Interaction**		
		F	W	S			C	S	C × S
Proteobacteria	1st	99.7Aa	78.2Aa	0.13Bb	9.34	0.0007	<0.0001	<0.0001	<0.0001
	2nd	94.9Aa	99.3Aa	3.19Bb	2.93	<0.0001			
	3rd	87.6Aa	98.0Aa	97.9Aa	5.87	0.4079			
	SEM	6.40	9.38	1.14					
	*p*-value	0.4560	0.2801	<0.0001					
Firmicutes	1st	0.063B	21.39B	99.80Aa	9.41	0.0007	<0.0001	<0.0001	<0.0001
	2nd	4.29B	0.65B	96.66Ab	2.59	<0.0001			
	3rd	11.85	1.05	0.28c	5.85	0.3584			
	SEM	6.33	9.42	0.87					
	*p*-value	0.4581	0.2799	<0.0001					
Actinobacteria	1st	0.1400	0.0033	0.0100	0.081	0.4528	0.0979	0.8543	0.6175
	2nd	0.5733	0.0333	0.0900	0.24	0.2988			
	3rd	0.4133	0.6767	1.0800	0.59	0.7333			
	SEM	0.32	0.37	0.41					
	*p*-value	0.6501	0.0400	0.2070					
Cyanobacteria	1st	0.0067	0.3667	0.0567	0.21	0.4607	0.4623	0.4402	0.2311
	2nd	0.0400	0.0000	0.0567	0.026	0.3480			
	3rd	0.0600	0.0233	0.6167	0.26	0.2800			
	SEM	0.037	0.21	0.26					
	*p*-value	0.6162	0.4252	0.2952					
Bacteroidetes	1st	0.1367	0.0000	0.0000	0.079	0.4219	0.8053	0.4116	0.5354
	2nd	0.1700	0.0033	0.0000	0.095	0.4094			
	3rd	0.0533	0.2067	0.0300	0.12	0.5506			
	SEM	0.12	0.12	0.012					
	*p*-value	0.7996	0.4116	0.2063					

Values are mean (*n* = 3) ± SE. Different capital letters differ among F, W, and S in the same cutting; Different lowercase letters differ among different cuttings *p* = 0.05. F, fresh alfalfa; W, wilted alfalfa; S, ensiled alfalfa; 1st, the first cutting; 2nd, the second cutting; 3rd, the third cutting.

**Figure 4:**
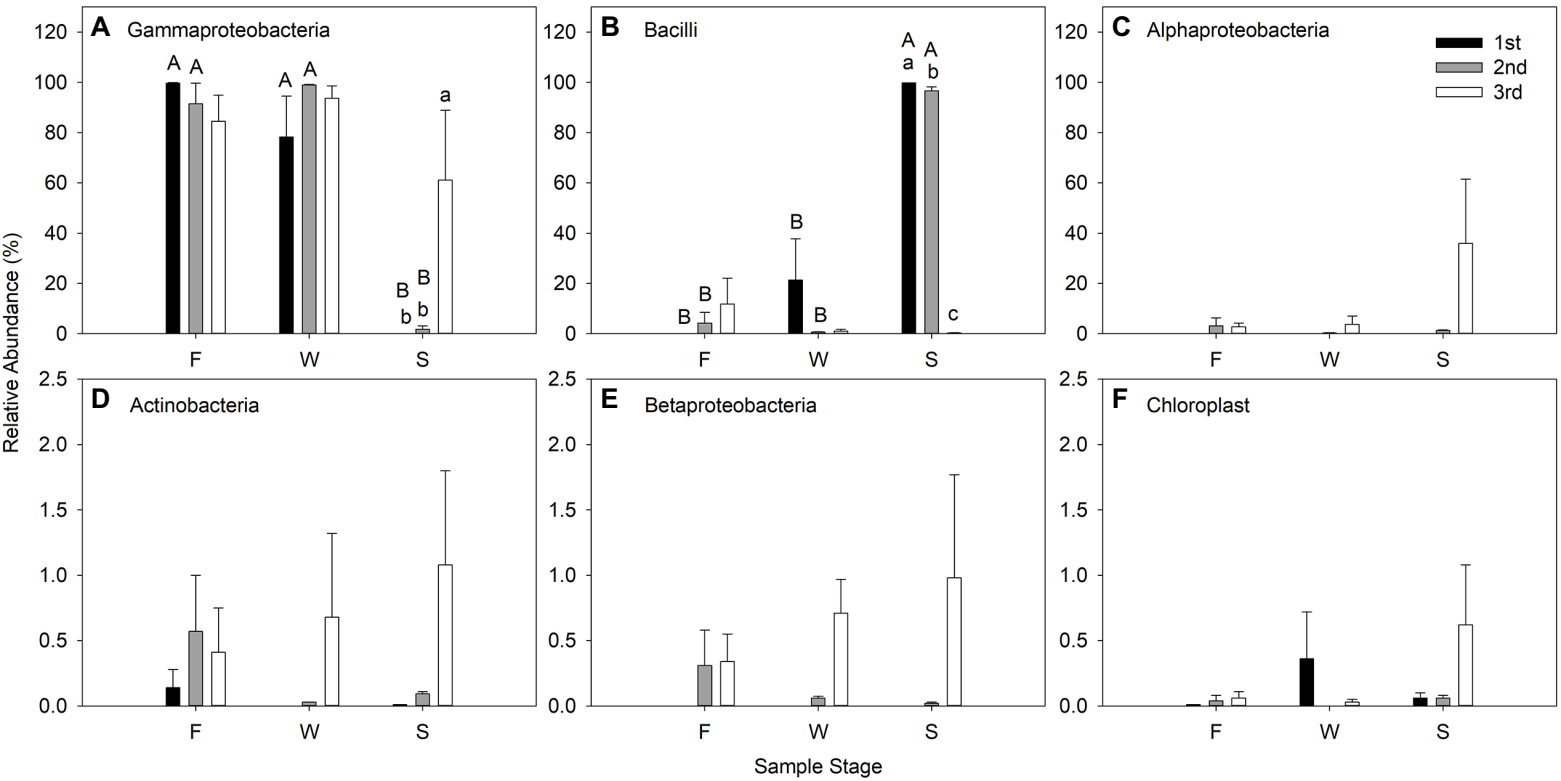
Relative abundance (% of individual taxonomic group) of dominant bacteria classes (mean ± SE, *n* = 3) in microbial communities following fresh alfalfa (F), wilted alfalfa (W) and ensiled alfalfa (S) at different cuttings. The black filled bar indicate the first cutting alfalfa, the gray filled bar indicted the second cutting alfalfa, and the non-filled bar indicate the third cutting alfalfa. Bar with different capital letters differ among F, W, and S in the same cutting; Bar with different lowercase letters differ among different cuttings *p* = 0.05.

Gammaproteobacteria predominated in F and W of the three cuttings at class level; however, Bacilli were the most predominant bacteria in the alfalfa silage in the first and second cuttings, but not in the third cutting. Gammaproteobacteria and Alphaproteobacteria predominated in the alfalfa silage the third cutting. Wilting did not affect the relative abundance of bacteria at the class level compared with F; however, ensiling significantly decreased the relative abundance of Gammaproteobacteria and increased Bacilli in the first and second cuttings (Figure 4).

**Figure 5:**
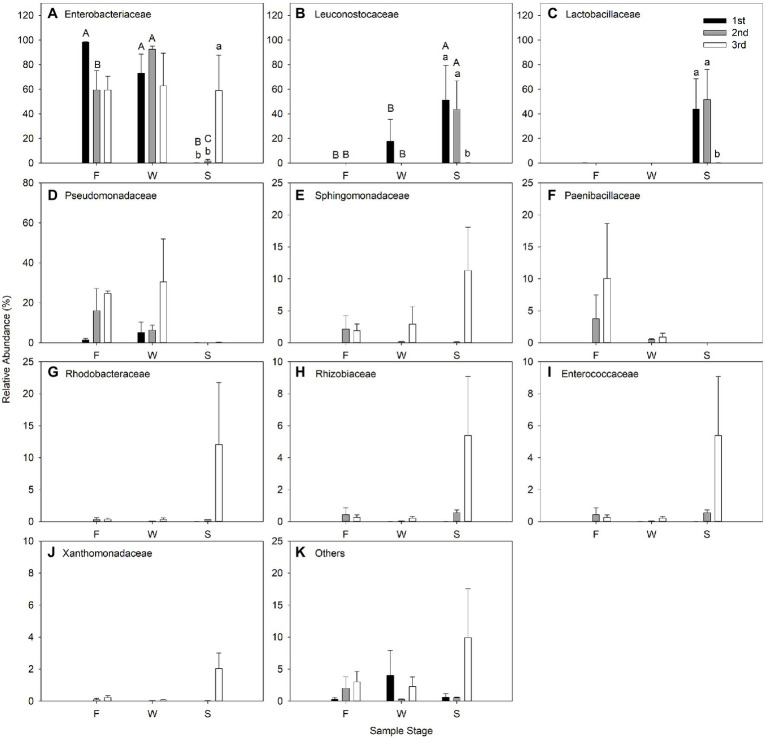
Relative abundance (% of individual taxonomic group) of dominant bacteria family (mean ± SE, *n* = 3) in microbial communities following fresh alfalfa (F), wilted alfalfa (W) and ensiled alfalfa (S) at different cuttings. The black filled bar indicate the first cutting alfalfa, the gray filled bar indicted the second cutting alfalfa, and the non-filled bar indicate the third cutting alfalfa. Bar with different capital letters differ among F, W, and S in the same cutting; Bar with different lowercase letters differ among different cuttings *p* = 0.05.

The effect of cutting time and sampling stage on relative abundance of bacteria at family level are shown in Figure 5. Enterobacteriaceae predominated in all cuttings of F and W, and comprised the most bacteria in alfalfa silage in the third cutting. Leuconostocaceae and Lactobacillaceae were the two predominant families in alfalfa silage in the first and second cuttings (Figure 5).

### Comparisons at the genus level

**Figure 6:**
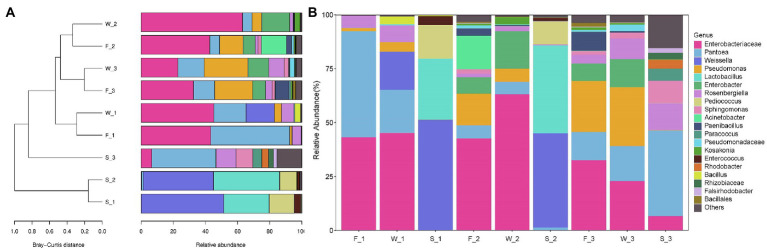
Clustering of samples **(A)** and relative abundance of bacterial communities in alfalfa at genera level **(B)**. F, fresh alfalfa; W, wilted alfalfa; S, ensiled alfalfa; 1, the first cutting; 2, the second cutting; 3, the third cutting. Bray–Curtis similarity index was calculated using the relative abundance of 20 most predominant bacterial communities at genera level, and hierarchical clustering was calculate using distance matrix using Qiime.

The Bray–Curtis similarity index was used to identify the differences in alfalfa bacterial community composition across three cuttings and three sampling stages (Figure 6A). At genus level, bacterial community in the first cutting in F (F_1) and in the first cutting in W (W_1), in the second cutting in F (F_2) and in the second cutting in W (W_2), and in the third cutting in F (F_3) and in the third cutting in W (W_3) clustered closely together, indicating that bacterial community composition was similar between F and W. F_2, W_2, F_3 and W_3 clustered closely together, and were distinct from F_1 and W_1, indicating that the bacterial community composition was similar between the second cutting and the third cutting, and dissimilar to the first cutting. The bacterial community composition of all F and W was distinct from S, the first cutting in S(S_1), the second cutting in S(S_2) and the third cutting in S (S_3) were distinct from each other.

Cutting time and sampling stage effects on the percentage distribution of the 20 most predominant genera are shown in Figure 6B. *Enterobacteriaceae_unclassified*, *Pantoea*, *Pseudomonas*, and *Rosenbergiella* were found in F in all three cuttings. *Pantoea* (49.19%) and *Enterobacteriaceae_unclassified* (43.29%) predominated in F_1; *Enterobacteriaceae_unclassified* (42.69%), *Acinetobacter* (15.59%), *Pseudomonas* (14.65%) and *Pantoea* (6.12%) predominated in F_2; and *Enterobacteriaceae_unclassified* (32.61%), *Pseudomonas* (23.63%) and *Pantoea* (13.11%) predominated in F_3. Wilt treatment increased the relative abundance of *Enterobacteriaceae_unclassified* in the first and second cuttings; increased *Enterobacter* in all cuttings; decreased the relative abundance of *Pantoea* in the first and second cuttings; and decreased *Paenibacillus* in the second and third cuttings. The relative abundance of *Enterobacteriaceae_unclassified* in W_1 (45.22% vs. 43.29%) and W_2 (63.12% vs. 42.69%) was higher than in F_1 and F_2, respectively, but in W_3 was lower than in F_3 (22.89% vs. 32.16%). The relative abundance of *Pantoea* in W_1 (20.02% vs. 49.19%) and W_2 (5.86% vs. 6.12%) was lower than in F_1 and F_2, respectively, but *Pantoea* in W_3 was higher than in F_3 (16.27% vs. 13.11%).

The bacterial community of ensiled alfalfa was significantly affected by cutting times. *Weissella* (43.64–51.29%), *Lactobacillus* (28.31–40.85%), *Pediococcus* (10.58–15.59%) and *Enterococcus* (1.39–4.09%) predominated in alfalfa silage in the first and second cuttings. However, *Pantoea* (39.75%), *Rosenbergiella* (12.46%), *Sphingomonas* (10.30%) and *Enterobacteriaceae_unclassified* (6.64%) predominated in the third cutting.

### Chemical composition and microbial population of fresh, wilted, and ensiled alfalfa

Table 2 shows the chemical composition and microbial populations of F, W and S in all three cuttings. The DM of alfalfa was significantly influenced by both cutting time and wilting. Wilting increased alfalfa DM; however, ensiling did not change the DM of alfalfa, except in the first cutting. The DM of F was similar between the second and the third cutting, but the DM of W in the third cutting was higher than in the second cutting. The DM of S differed significantly among the three cuttings, and the order from high to low was: third cutting > second cutting > first cutting. CP did not change among F, W and S, but was influenced by cuttings: the CP of the third cutting was significantly higher than in the first cutting. The WSC content of alfalfa was significantly influenced by both cuttings and wilting; wilting increased WSC content by 54.9, 47.6 and 53.0%, respectively, compared with F in the three cuttings, and ensiling decreased the WSC content by 54.3, 54.4 and 52.0%, respectively, compared with W in the three cuttings. The WSC content of the third cutting was higher than in the other two cuttings. The NDF was changed not by wilting, but by ensiling. Compared with W, the NDF decreased by 20.5, 42.2 and 29.7%, respectively, in S in the three cuttings. The NDF in the first and second cuttings was higher than in the third cutting. A similar tendency was found in ADF compared with NDF. Wilting increased the number of LAB, but not significantly compared with F; the number of LAB in S was higher than in W; and the number of LAB in F, W, or S in the second cutting was highest of all three cuttings. Wilting increased the number of yeast and the greatest numbers were found in the third cutting. Wilting significantly decreased the number of CB in the third cutting, but not in the first and the second cuttings. The numbers of CB in S were significantly lower than those of F and W.

**Table 2:** Characteristics of chemical and microbial counts of fresh, wilted, and ensiled alfalfa at three cuttings.

**Items**	**Cutting**	**Stages**			**SEM**	***p*-Value**	**Interaction**		
		F	W	S			C	S	C × S
DM (g/kg FM)	1st	229.3Cc	426.9Bc	439.4Ac	1.63	<0.0001	0.0088	<0.0001	0.0051
	2nd	271.9Ba	471.3Ab	485.4Ab	4.93	<0.0001			
	3rd	244.0Bb	616.7Aa	604.1Aa	4.28	<0.0001			
	SEM	1.57	5.09	3.46					
	*p*-value	<0.0001	<0.0001	<0.0001					
CP (g/kg DM)	1st	189.7b	177.2b	189.3b	6.81	0.3920	<0.0001	0.8151	0.1389
	2nd	199.1ab	214.9a	200.3ab	6.08	0.1581			
	3rd	213.6a	219.5a	220.3a	4.49	0.5495			
	SEM	5.48	5.73	6.32					
	*p*-value	0.0566	0.0037	0.0281					
Ash (g/kg DM)	1st	101.6	98.5b	110.3	4.07	0.1857	0.2341	0.5904	0.0727
	2nd	112.4A	105.3Ba	101.9B	2.11	0.0198			
	3rd	98.1	101.2ab	103.4	4.62	0.7254			
	SEM	4.62	1.52	4.07					
	*p*-value	0.1523	0.0499	0.3271					
WSC (g/kg DM)	1st	41.44Bb	64.20Aab	29.35C	3.2983	0.0008	<0.0001	<0.0001	0.4673
	2nd	33.64Bc	49.65Ab	22.64C	3.6418	<0.0001			
	3rd	49.31Ba	75.42Aa	36.15B	4.838	0.0033			
	SEM	1.8836	4.5733	3.3223					
	P-value	0.0032	0.0204	0.0743					
NDF (g/kg DM)	1st	461.5Aab	464.5A	369.5Ba	7.75	0.0002	<0.0001	<0.0001	0.1944
	2nd	486.0Aa	491.9A	372.9Ba	7.04	<0.0001			
	3rd	439.3Ab	456.3A	320.9Bb	9.27	<0.0001			
	SEM	7.64	8.71	7.82					
	P-value	0.0143	0.0620	0.0057					
ADF (g/kg DM)	1st	358.7Aa	361.1Aa	270.6Bb	7.63	0.0002	<0.0001	<0.0001	0.3842
	2nd	374.9Aa	379.1Aa	319.2Ba	9.21	0.0064			
	3rd	280.1Ab	287.2Ab	226.7Bc	12.99	0.0317			
	SEM	6.15	12.74	10.56					
	*p*-value	<0.0001	0.0049	0.0025					
LAB (log _10_ cfu/g of FM)	1st	<2Bc	<2Bb	5.927Ab	0.21	<0.0001	<0.0001	<0.0001	<0.0001
	2nd	3.11Ba	3.63Ba	7.16Aa	0.18	<0.0001			
	3rd	2.74Bb	3.19Ba	4.57Ac	0.23	0.0035			
	SEM	0.098	0.16	0.25					
	*p*-value	0.0006	0.0010	0.0005					
Yeast (log _10_ cfu/g of FM)	1st	<2Bc	<2Bc	5.06Ab	0.44	0.0013	<0.0001	<0.0001	<0.0001
	2nd	3.53Bb	3.04Bb	6.96Aa	0.19	<0.0001			
	3rd	4.50Aa	3.80Ba	4.69Ab	0.18	0.0263			
	SEM	0.055	0.17	0.38					
	*p*-value	<0.0001	0.0009	0.0080					
Coliform bacteria (log _10_ cfu/g of FM)	1st	2.74Ab	3.21Ac	<2Bb	0.26	<0.0001	<0.0001	<0.0001	<0.0001
	2nd	6.77Aa	6.54Aa	4.44Ba	0.32	0.0014			
	3rd	6.57Aa	5.31Bb	<2Cb	0.17	<0.0001			
	SEM	0.12	0.33	0.23					
	*p*-value	<0.0001	0.0011	<0.0001					
Aerobic bacteria (log _10_ cfu/g of FM)	1st	3.45Bb	3.27Bb	6.55Aa	0.17	<0.0001	<0.0001	0.0008	<0.0001
	2nd	7.02Aa	6.88Aa	6.75Aa	0.23	0.6925			
	3rd	7.06Aa	6.44Ba	4.91Cb	0.14	0.0001			
	SEM	0.12	0.19	0.20					
	*p*-value	<0.0001	<0.0001	0.0006					

Values are mean (*n* = 3) ± SE. Different capital letters differ among F, W, and S in the same cutting; Different lowercase letters differ among different cuttings *p* = 0.05. F, fresh alfalfa; W, wilted alfalfa; S, ensiled alfalfa; 1st, the first cutting; 2nd, the second cutting; 3rd, the third cutting. DM, dry matter; CP, crude protein; WSC, water soluble carbohydrates; NDF, Neutral detergent fiber; ADF, acid detergent fiber; LAB, lactic acid bacterial.

### Fermentation quality of ensiled alfalfa in three different cuttings

The fermentation properties of alfalfa silage produced by cutting at different times are shown in Table 3. There were significant differences in the pH levels, as well as in LA, AA, PA and BA, depending on the cutting time. The pH of the silage ranged from 5.59 to 5.72, with the silage produced by the first cutting and the third cutting having the lowest and highest pH levels, respectively. The LA content of all alfalfa silage ranged from 11.39 to 28.78 g/kg DM with the second cutting having the highest LA content and the third cutting the lowest. The AA content of the alfalfa silage ranged from 1.14 to 8.18 g/kg DM, with the silage samples produced by the first cutting and the second cutting having the highest and lowest levels, respectively. The PA content of natural fermentation alfalfa silage samples ranged from 4.16 to 8.19 g/kg DM; the highest PA content was found in the first cutting, and the lowest in the third cutting. The BA content of all alfalfa silage samples ranged from 0 to 19.13 g/kg DM; the silage from the third cutting sample had the highest BA content and the first cutting sample had the lowest. The ammonia-N content level among the three cuttings showed no significant differences.

**Table 3:** Fermentation quality of alfalfa 60d after ensiling at three cuttings.

Item	1st	2nd	3rd	SEM	*p*-Value
pH	5.59 ± 0.01b	5.69 ± 0.04a	5.72 ± 0.02a	0.022	0.046
LA (g/kg DM)	19.86 ± 2.50ab	28.78 ± 3.22a	11.39 ± 5.06b	4.026	0.046
AA (g/kg DM)	8.18 ± 0.99a	1.14 ± 0.26b	4.53 ± 2.22ab	1.154	0.041
PA (g/kg DM)	8.19 ± 0.98a	4.46 ± 0.58b	4.16 ± 0.64b	0.652	0.016
BA (g/kg DM)	0c	6.16 ± 3.09b	19.13 ± 2.74a	2.649	0.0035
NH_3_-N (g/kg TN)	198.5 ± 0.27	218.8 ± 1.09	225.9 ± 0.40	4.664	0.0069

Values are mean (*n* = 3) ± SE. Different lowercase letters differ among different cuttings *p* = 0.05. 1st, the first cutting; 2nd, the second cutting; 3rd, the third cutting. LA, lactic acid; AA, acetic acid; PA, propionic acid; BA, butyric acid; NH3-N, ammonia-N; DM, dry matter; TN, total nitrogen.

### Correlations between relative abundance of bacteria, fermentation quality indices, and DM

**Figure 7:**
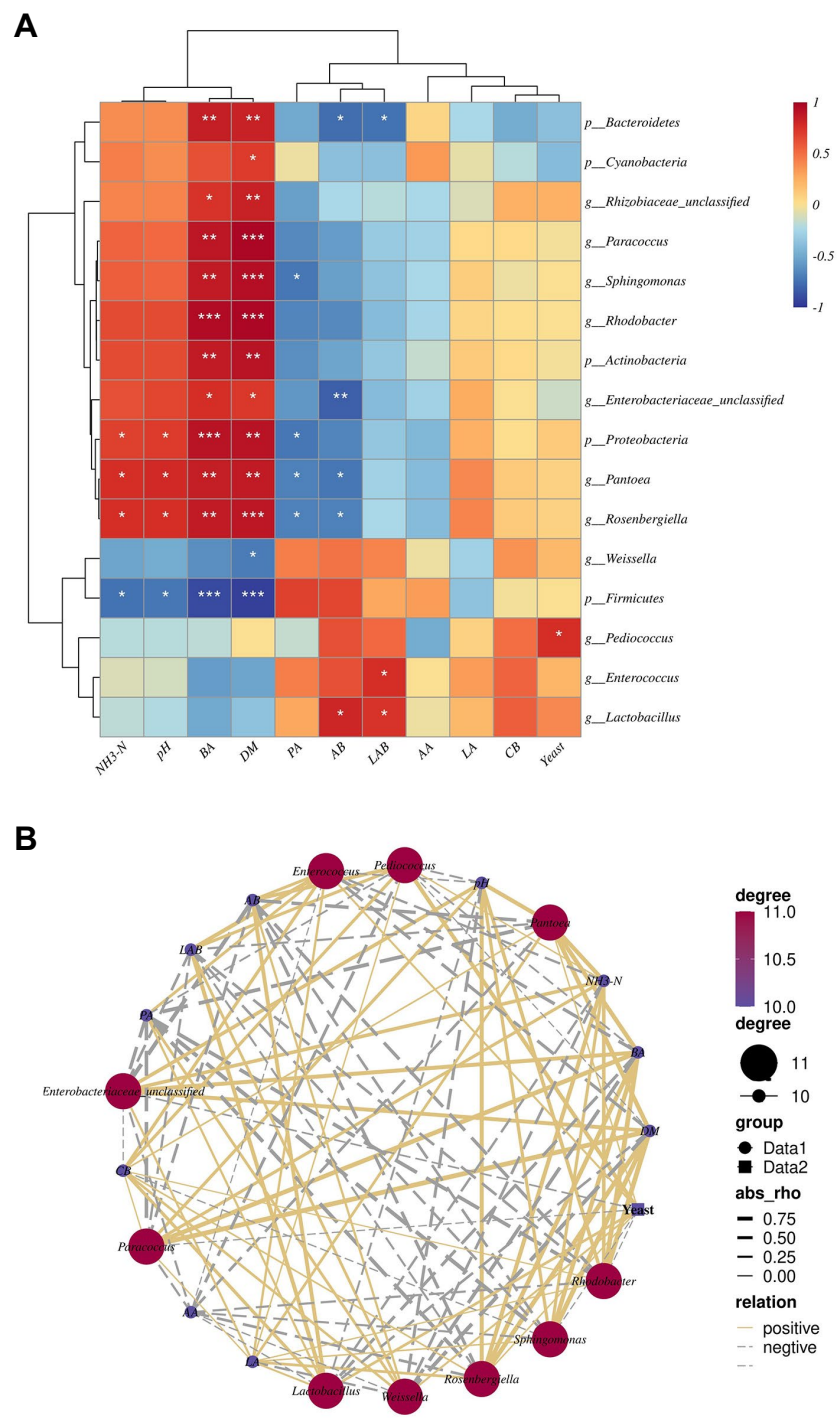
**(A)** Spearman correlation heatmap of the top tenth bacterial phylum, genus, and fermentation properties. **(B)** Correlation network among main bacterial genera top tenth and fermentation properties. The values presented by colors in the heat map correspond to the Spearman correlation coefficient r, which ranged between −1 and 1, where *r* < 0 indicates a negative correlation(blue), *r* > 0 indicates a positive correlation (red), “*” represents *p* < 0.05, “**” represents *p* < 0.01, “***” represents *p* < 0.001. In(b), absolute value of correlation coefficient > 0.5 and *p* < 0.05. LA, lactic acid; AA, acetic acid; PA, propionic acid; BA, butyric acid; DM, dry matter; CB, number of coliform bacteria; LAB, number of lactic acid bacterial; Yeast, number of yeast; AB, number of Aerobic bacteria.

Spearman’s correlation analyses of fermentation properties with abundance of the 10 most abundant phyla and genera in the three cuttings of alfalfa silage are shown in Figure 7. *Lactobacillus* (*r* = 0.75) and *Enterococcus* (*r* = 0.77) were positively associated with number of LAB; *Sohingomomas* (r = −0.73), *Pantoea* (*r* = −0.69) and *Rosenbergiella* (*r* = −0.68) were negatively associated with PA; and *Pantoea* (*r* = 0.78) and *Rosenbergiella* (*r* = 0.77) were positively associated with pH and NH_3_-N. *Rhodobacter* (*r* = 0.96 and *r* = 0.97), *Sphingomomas* (*r* = 0.87 and *r* = 0.93), *Paracoccus* (*r* = 0.90 and *r* = 0.97), *Enterobacteriaceae_unclassified* (*r* = 0.77 and *r* = 0.73), *Pantoea* (*r* = 0.50 and *r* = 0.53) and *Rosenbergiella* (*r* = 0.87 and *r* = 0.90) were positively associated with BA and DM. *Proteobacteria* were positively associated with NH_3_-N (*r* = 0.73), pH (*r* = 0.76), BA (*r* = 090), and DM (*r* = 0.93) (Figure 7A). NH_3_-N and pH had a positive correlation with *Enterobacteriaceae_unclassified* (*r* = 0.69 and *r* = 0.66), *Paracoccus* (*r* = 0.77 and *r* = 0.73), *Rosenbergiella* (*r* = 0.72 and *r* = 0.78), *Sphingomonas* (*r* = 0.56 and *r* = 0.55), *Rhodobacter* (*r* = 0.65 and *r* = 0.66) *and Pantoea* (*r* = 0.79 and *r* = 0.76), but a negative correlation with *Lactobacillus*, *Enterococcus*, *Pediococcus*, and *Weissella* (Figure 7B).

### Functional of bacterial communities in ensiled alfalfa in three cuttings

**Figure 8:**
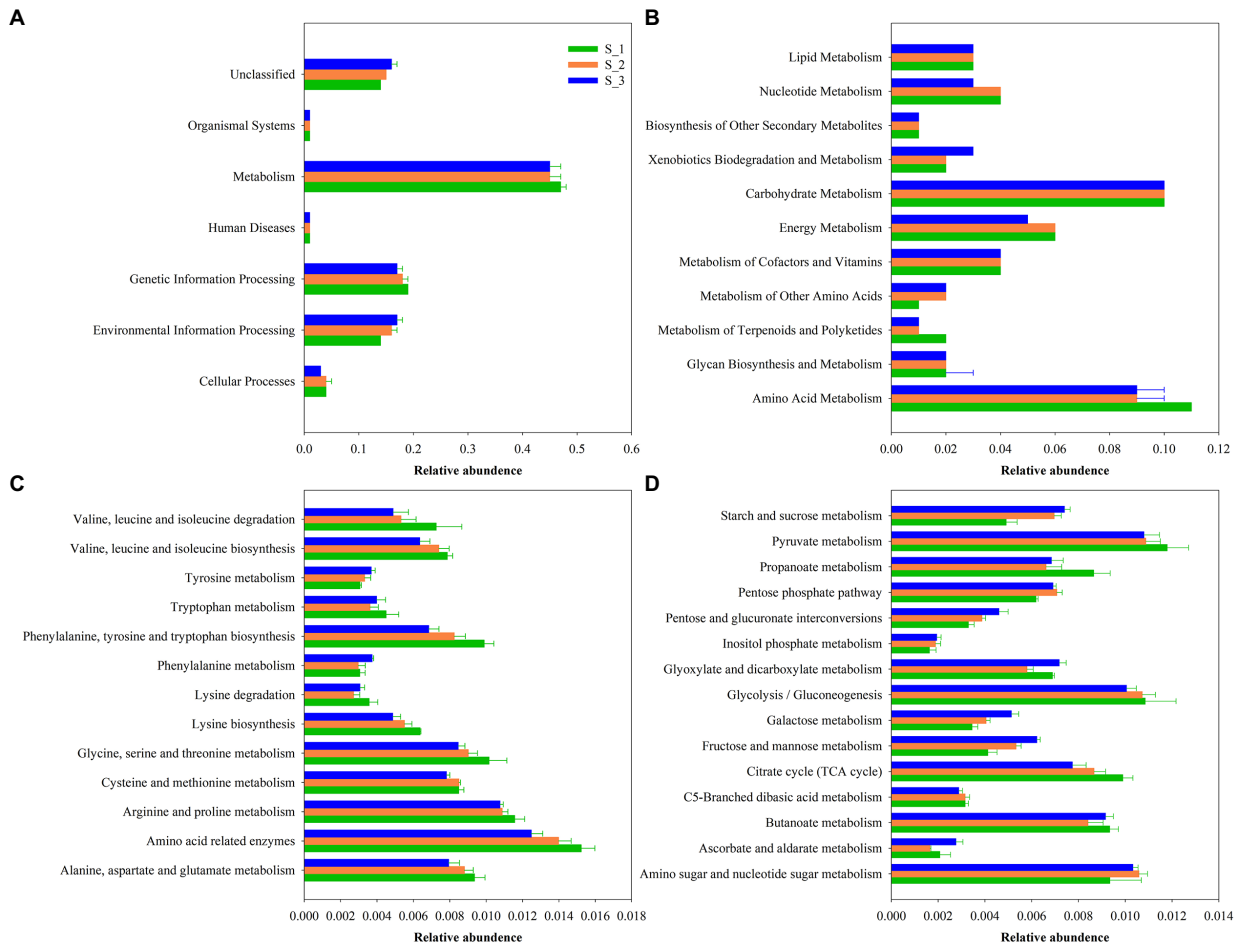
Bar graphs showing 16S rRNA gene-predicted functional profiles on the first **(A)**, second **(B)** pathway level, amino acid metabolism, **(C)** and carbohydrate metabolism **(D)** of the third pathway level obtained with Tax4Fun. S_1, ensiled alfalfa in the first cutting, S_2, ensiled alfalfa in the second cutting, S_3, ensiled alfalfa in the third cutting. The green filled bar indicate S_1, the orange filled bar indicate S_2, the blue filled bar indicate S_3.

The 16S rRNA gene-predicted functional profiles on the first (Figure 8A) and second (Figure 8B) pathway levels, amino acid(Figure 8C), and carbohydrate (Figure 8D) metabolism are described in Figure 8. As shown in Figure 8A, the relative abundance of “Metabolism” was obviously higher than other pathway. As shown in Figure 8B, the relative abundances of amino acid and carbohydrate metabolism were much higher than other pathway. The amino acid metabolism in S_3 was higher than in S_1 and S_2. As seen in Figure 8C, the amino acid metabolism including valine, leucine and isoleucine biosynthesis and degradation, phenylalanine, tyrosine and tryptophan biosynthesis, Lysine biosynthesis, glycine, serine, and threonine metabolism, arginine and proline metabolism, amino acid related enzymes, alanine, aspartate and glutamate metabolism were the highest in the S_1 and lowest in S_3.Tyrosine, tryptohan, phenylalanine metabolism were highest in S_3 and lowest in S_1. As illustrated in Figure 8D, the carbohydrate metabolism including pyruvate, propanoate, glycolysisi/gluconeogenesis, cirtrate cycle(TCA cycle) were highest in S_1, and lowest in S_3. Starch and sucrose, pentose phosphate, pentose, and glucuronate interconversion, inositol phosphate, galactose, fructose and mannose metabolism were highest in S_3 and lowest in S_1.

**Figure 9:**
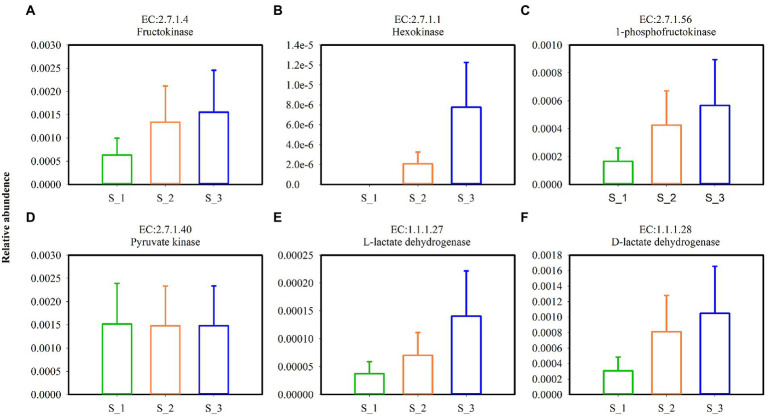
Key enzymes involved in glycolytic pathway **(A–D)** and lactate dehydrogenase **(E,F)** in the three cutting alfalfa silage. S_1, ensiled alfalfa in the first cutting, S_2, ensiled alfalfa in the second cutting, S_3, ensiled alfalfa in the third cutting. The green non-filled bar indicate S_1, the orange non-filled bar indicate S_2, the blue non-filled bar indicate S_3. EC, reference metabolic pathway highlighting numbers; means within the same column with different letters differ significantly from each other (*p* < 0.05).

**Figure 10:**
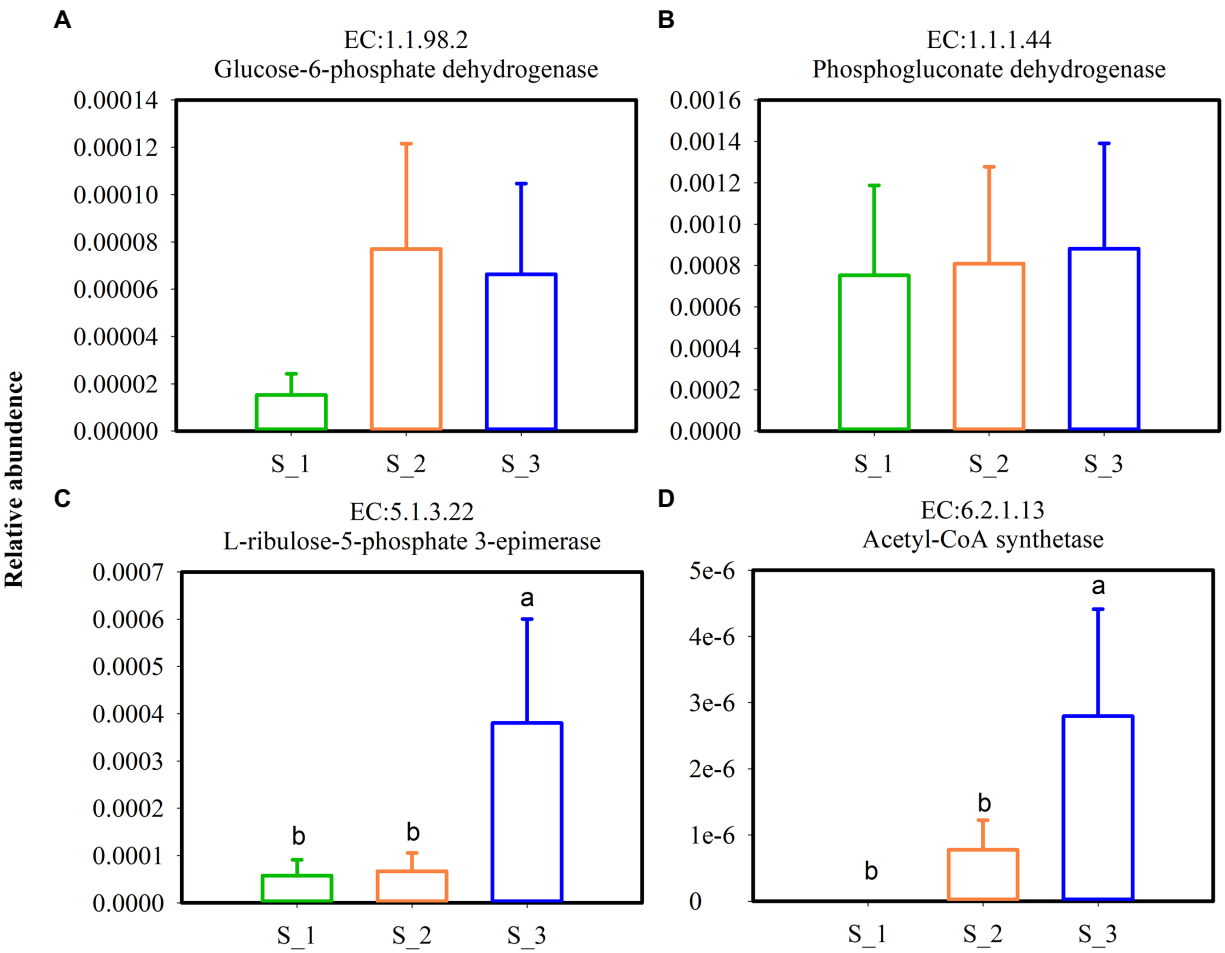
Key enzymes involved in pentose phosphate pathway **(A–C)** and acetyl-CoA synthetase **(D)** in the three cutting alfalfa silage. S_1, ensiled alfalfa in the first cutting, S_2, ensiled alfalfa in the second cutting, S_3, ensiled alfalfa in the third cutting. The green non-filled bar indicate S_1, the orange non-filled bar indicate S_2, the blue non-filled bar indicate S_3. EC, reference metabolic pathway highlighting numbers; means within the same column with different letters differ significantly from each other (*p* < 0.05).

Key enzymes involved in the glycolytic pathway (Figures 9A–D) and lactate dehydrogenase (Figures 9E,F) in alfalfa silage are shown in Figure 9. Except pyruvate kinase, the relative abundances of fructokinase, hexokinase, 1-phosphofructokinas, L-lactate dehydrogenase, and D-lactate dehydrogenase were highest in S_3 and lowest in S_1. Key enzymes involved in pentaose phosphatepathway (Figures 10A–C) and acetyl-CoA synthetase (ACS) (Figure 10D) in ensiled alfalfa are found in Figure 10. Glucose-6-phosphate dehydrogenase was lowest in S_1, however, L-ribulose-5-phoshate3-epimerase and acetyl-CoA synthetase were highest in S_3.

## Discussion

This experiment was conducted as part of a broader study aimed at understanding the microbial community and nutritional changes among F, W and S in three cuttings per year.

### Chemical composition and microbial populations of fresh, wilted, and ensiled alfalfa among three cuttings

The moisture content of alfalfa plays an important role in the fermentation process. It has been reported that the optimal DM content of W to prevent effluent production is 300–400 g/kg FW (Duniere et al., 2013). The increase in DM content after wilting in our study was similar to that observed in previous studies (Thi Minh et al., 2018; Agarussi et al., 2019a). The DM of W, however, was significantly different among the three cuttings, and especially in the third cutting (Table 2). The harvest time and drying time were same in all three cuttings, but the DM of W in the third cutting was far higher than in the first and second cuttings, mainly because of the different weather characteristics. During the third-cutting harvest, the stronger wind removed a considerable amount of moisture, leading to a higher DM content.

Water-soluble carbohydrates (WSC) are an important fermentation substrate for LAB. Generally, 60–70 g/kg DM WSC is necessary for well-preserved alfalfa ensilage (Smith, 1962). In our study, the WSC content of F in all three cuttings was below 60 g/kg DM. Apart from the second cutting, the WSC content of W in the first and the third cuttings was higher than 60 g/kg DM, suggesting that wilting can increase alfalfa WSC content. A similar result was found in other plant research (Gao et al., 2021); however, some previous research has shown a reduction in WSC concentration after wilting (Tao et al., 2017; Agarussi et al., 2019a; Yang et al., 2022). As we expected, there was an increase in WSC after wilting in all three cuttings, at levels of approximately 54.9, 47.6 and 53.0%, respectively, compared with F. Generally, the WSC content in the parent forage should be >2.5% on a fresh forage basis, for good silage fermentation (Kaiser et al., 2004). The WSC contents on a fresh forage basis in F_1, F_2 and F_3 were 0.95, 0.91, and 1.20%, respectively. The WSC contents on a fresh forage basis in W_1, W_2, and W_3 were 2.74, 2.34, and 4.65%, respectively, indicating that wilting is essential for alfalfa silage to increase WSC content. This finding was not, however, consistent with previous studies that found a reduction of approximately 7% in WSC after the wilting process (Rangrab et al., 2000; Agarussi et al., 2019a). In all three cuttings the NDF was decreased not by wilting, but by ensiling, as described in a previous study (Sun et al., 2021), and a similar tendency was found in ADF.

The epiphytic LAB of fresh material is also a crucial factor: more than 5 lg cfu/g FM is required to obtain good fermentation (Cai et al., 1998). The LAB number of F was far below the value in all three cuttings, and the LAB number of F in the second cutting was the highest; we found that wilting increased the LAB number. In the first cutting neither LAB number nor undesirable microorganism numbers were relatively low, they were but relatively high in both second and third cuttings. Our results suggested that wilting increased the number of LAB and decreased the number of undesirable microorganisms. The wilting process increased the forage LAB population from 5.28 lg cfu/g to 6.22 lg cfu/g, similar to the result reported by Agarussi et al. (2019a). A previous study has shown that wilting for 6 h led to LAB, yeast, mold and CB values of 6.54, 5.19, 5.16, and 6.37, respectively (Agarussi et al., 2019b). The fermentation process increased the number of LAB and decreased the number of CB, but did not inhibit yeast. The higher populations of yeast in alfalfa silage at the end of fermentation was probably because some yeasts can grow in anaerobic and acidic environments (Santos et al., 2015).

### Fermentation quality of alfalfa among three cuttings

The pH level provides a basic value for evaluating silage fermentation. To obtain silage of an excellent quality, a large enough LA-producing bacterial population is required, to induce a rapid drop in pH (Kerstin et al., 2017). Well-fermented silage should have a pH of 4.2 or lower (Edwards and McDonald, 1978). In the present study no LAB additives were used, and the results showed that all cuttings of silage had a pH of higher than 4.2, suggesting a lower quality of natural fermentation in all three cuttings. The higher pH in alfalfa silage in this study, not only due to the high buffer capacity and low WSC content, but also maybe the wilting, the increased silage pH by wilting agree with previous studies (Gordon et al., 1999; Hashemzadeh-Cigari et al., 2011). pH values in all three cuttings were considerably above the ideal level (< 4.20), suggesting that natural alfalfa LA fermentation with no LAB addition was insufficient to stabilize the ensiled mass. The insufficient LA fermentation might have been due to insufficient levels of LAB and the high buffer capacity (BC) of the W. Similar results were found in a previous study (Yang et al., 2020). The AA concentrations differed significantly among the three cuttings, being highest in the first cutting and lowest in the second. AA is a promoter of aerobic stability during the ensiling process (Schmidt and Kung, 2010) and an effective inhibitor of fungi (Le Lay et al., 2016). The enhancement in AA accumulation may help in reducing high levels of microbes in S (Yang et al., 2020): our study showed a higher AA content and a lower microbial richness in the first cutting of alfalfa silage. The PA contents (8.19, 4.16, and 4.46 for the three cuttings, respectively) at the end of the fermentation period were within the acceptable range for this acid which, according to Mahanna (1993), should range from 1 to 10 g/kg DM in good-quality silage; similar results were found in an alfalfa silage study (Agarussi et al., 2019a). A good fermentation requires BA levels of less than 5 g/kg DM (Kung et al., 2018), alfalfa harvest at the third cutting was typical clostidial fermentation of ensiling, as evidenced by the decreased in lactic acid content and increased in butyric acid content, the results aggree with previous study of alfalfa silage with no-additive (Li R. et al., 2020).

The NH_3_-N concentrations in all three cuttings alfalfa of silage ranged from 198.5 to 225.9 g/kg TN. Less than 150 g/kg TN are considered to be acceptable for good fermentation in legume silage (Mahanna and Chase, 2003). The increased NH_3_-N concentrations in all three cuttings of alfalfa silage resulted from intense proteolysis during fermentation when sufficiently acidic conditions did not occur. Most plant proteolytic enzymes in alfalfa silage show greater activities at pH 5.0–6.0 (Tao et al., 2012), suggesting that the natural fermentation quality with no LAB in the study area were unacceptable. The results differed from a previous study which showed that W silage had low NH_3_-N concentrations (134.7 k/kg TN), perhaps as a result of different climatic conditions leading to low levels of BC arising during fermentation when sufficiently acidic conditions occurred (Agarussi et al., 2019a). The effects of *Clostridia* and plant proteolytic enzymes may be typical causes of NH_3_-N accumulation (Kung and Shaver, 2001).

### Bacterial diversity and composition of fresh, wilted, and ensiled alfalfa among three cuttings

Next-generation sequencing has been widely used in forage silage (Ogunade et al., 2018; Wang et al., 2022) to detect bacterial community composition and abundance, or to monitor the change in a bacterial community during the process of silage fermentation. This study revealed the relative abundance and diversity of bacteria in alfalfa at the fresh, wilting and silage stages in three different cuttings. The Chao1 index and observed_species (Figures 2A,B) were similar between F and W, and higher than S. PCoA and the Bray–Curtis similarity index were used to analyze beta diversity, and to identify the differences in alfalfa bacterial community composition across different stages of different cuttings. At the OTU level, both analyses showed that F and W of the second and the third cuttings clustered closely together, indicating that their bacterial community compositions were similar. The F and W of the first cutting clustered together, and were distinct from F and W of the second and third cuttings. This indicated that the bacterial community composition of F and W of the first cutting differed from that of the second and the third cuttings. When there was no clear separation between the bacterial communities in F and W, this indicated there was no change after wilting in all three cuttings. The clear separation between bacterial communities of the alfalfa silage and W (and F) indicated a shift after ensiling, consistent with previous studies (Ni et al., 2017a,b; Yang et al., 2019). Values in the S of the three cuttings were distinct from each other, showing that their bacterial communities differed from each other. The bacterial community structure of W_2 and W_3 were similar to each other, but bacterial community composition were separated after fermentation, perhaps because of the different storage temperatures between the second and the third alfalfa in the fermentation process. A previous study proved that storage temperature is the main environmental factor that influences the fermentation quality and microbial community of silage (Bai et al., 2022). The higher indices of Chao1 index and OTUs in F and W than in S in all three cuttings indicated that a more abundant bacterial community existed in the epiphytic bacteria in F and W compared with S. This is because, when a silo is sealed, the internal environment shifts from aerobic to anaerobic conditions and anaerobic microorganisms adapt and grow well, leading to a noticeable decrease in bacterial diversity and numbers during fermentation. Similar results were found in a previous study (Wang et al., 2022). In this study, the highest indices of OTUs, Shannon and Chao1 index in the third cutting, and the lowest in the first cutting, indicated that the most abundant bacterial community was present in the third cutting and the bacterial community with the lowest abundance was present in the first cutting, showing that microbial abundance differed among the three cuttings.

The bacterial species that are predominant is an important factor which can influence the silage fermentation process. Hence, analyzing the changes in bacterial composition during wilting and fermentation contributes to understand the ensiling process and promoting fermentation quality. The majority of the 16S rNDA sequences obtained from the F were associated with the phylum Proteobacteria (99.6%), and with the genera *Pantoea* (49.19%), *Enterobacteriaceae_unclassified* (43.29%), *Rosenbergiella* (5.66%), and *Pseudomonas* (1.43%) in the first cutting. In the second cutting the F were associated with the phylum *Proteobacteria* (94.93%) and *Firmicutes* (4.29%), and with the genera *Enterobacteriaceae_unclassified* (42.69%), *Acinetobacter* (15.59%), *Pseudomonas* (14.65%), *Enterobacter* (7.53%) and *Pantoea* (6.13%). In the third cutting they were associated with the phyla Proteobacteria (87.62%) and Firmicutes (11.85%), and with the genera *Enterobacteriaceae_unclassified* (32.61%), *Pseudomonas* (23.63%), *Pantoea* (13.11%), *Paenibacillus* (8.74%) and *Enterobacter* (8.31%). These results suggest that the structure of the bacterial population changed in the different cuttings. It is likely that the colonization of plant surfaces by bacteria is a complex process that is dependent on climate in different seasons. The most dominant phylum in F was Proteobacteria in the first or second cutting, and Firmicutes in the third cutting. This indicated that the epiphytic bacterial community on raw material is affected by cutting times, differing from the findings of Guo et al. (2018) and Wang et al. (2022), although in both of these studies the cutting times were not supplied. It may be that the epiphytic bacterial community on raw material is affected by climate and geographical location (Wang et al., 2020b).

Most studies show that the majority of the microbial communities related to LA fermentation in silage belong to the phylum Firmicutes and to the genera *Lactobacillus*, *Pedicoccus*, *Lactococcus*, *Weissella* and *Leuconostoc* (Pang et al., 2011). Our study revealed that approximately 99.8% of the bacterial community in first cutting of alfalfa silage and 96.7% in the second cutting belonged to the phylum Firmicutes, consisting mainly of class Bacilli, families Leuconostocaceae and Lactobacillaceae and genera *Weissella*, *Lactobacillus* and *Pediococcus*. *Weissella* remained in relatively higher abundance in the first and the second cuttings of alfalfa silage. This was consistent with the report of Wang et al. (2022) and inconsistent with that of Graf et al. (2016), who reported that *Weissella* was considered to be an early colonizer which was then replaced by acid-resistant *Lactobacilli* as fermentation progressed. In our study, *Weissella* domination in the first and second cuttings may have been because pH was high and *Weissella* was not replaced by acid-resistant LAB. Ogunade et al. (2018) assessed the bacterial community of alfalfa silage *via* the Illumina MiSeq platform and reported that the majority of genera detected were similar to our results in alfalfa silage in the first and second cuttings (Ogunade et al., 2018). Bao et al. (2016), however, assessed the bacterial community of alfalfa silage *via* single molecule real-time sequencing technology and found that the majority of genera detected were *Lactobacillus*, *Weissella*, *Pediococcus*, and *Pantoea*. NH_3_-N contents were higher in all three cuttings. An earlier study has proved that the existence of Enterobacteriaceae is undesirable during ensiling because members of this family complete with LAB for nutrients and production of NH_3_-N (Duniere et al., 2013). However, there were no *Enterobacteriaceae* in the first and the second cuttings, and lower relative abundance of *Enterobacteriaceae* in the third cutting was observed. It is possible that NH_3_-N was promoted in the initial fermentation stage, because of the higher relative abundance of *Enterobacteriaceae* in F and W in all three cuttings.

### Linkages between fermentation properties and microbial characteristics

Silage fermentation is an intricate biological process which involves various microorganisms, leading to large metabolite values that influence the fermentation quality (Zi et al., 2021). Recent studies have clarified an interaction between the bacterial community and silage fermentation properties (Mcallister et al., 2018). Mantel tests revealed a close correlation between pH, NH_3_-N, DM, PA, BA, and the numbers of LAB, AB and yeasts with bacterial community composition, in contrast to Yang et al. (2020). The present study showed a significant correlation between DM and microorganism abundance in alfalfa silage, although Yang et al. (2019) did not find the same correlation. To better reveal the relationships between fermentation properties and microbial kinetics during the ensiling of alfalfa, we used a Spearman correlation heatmap at the phylum and genus level (Figure 7). Spearman’s correlation analysis showed that some potential spoilage by phyla and genera in alfalfa silage was positively correlated with NH_3_-N and pH. The potential spoilage genera in our study were *Pantoea* and *Rosenbergiella*, members of the Enterobacteriaceae family (Walterson and Stavrinides, 2015; Yang et al., 2020). They are also members of the Proteobacteria phylum, and potential promoters of NH_3_-N formation during ensiling. Some members of the *Pantoea* genus are facultative anaerobes and can survive a period of fermentation.

### Functional of bacterial communities of ensiled alfalfa among three cuttings

Recently, KEGG pathway database with PICRUSt and orthology (KO) classification were used to predict the metabolic pathways of silages (Bai et al., 2021; Xu et al., 2021; Wang et al., 2022). ‘Metabolism’ was the predominant metabolic on the level 1 pathway (Figure 8A), which suggested that the fermentation process in silage is mediated by microbial activities through complicated metabolic pathways to degrade substrates or transform metabolites. Wang et al. (2022) reported that carbohydrates metabolism and amino acid metabolism were predominant metabolic pathways on the level 2 related to alfalfa silage fermentation. The same result were found in our study, therefore, the amino and carbohydrate metabolism were further investgated on the third pathway level (Figures 8C,D). Amino acid metabolism might relfect the capacity of the bacterial populations in the siliange to sythesize amino acid *de novo* (Keshri et al., 2019), amino metabolism in S_1 were highest, maybe due to the high relative abundance of LAB, since LAB do not synthesize all their essential amino acid, LAB relay on proteolytic systems to provide essential amino acid for their growth (Balamurugan and Udayakumar, 2019). Previous study showed carbohydrate metabolism were positively with relative abundances of total LAB (Bai et al., 2021), however, in present study, carbohydrate metabolism in the level_2 pathway were not differ among three cutting, while the relative abundance of LAB in first and second cutting were higher than in the third cutting. Because some undesirable microorganism competitive use of carbohydrates with LAB, like clostridium and Enterobacter. Carbohydrate metabolism in the level_3 pathway were differ among three cutting. Propanoate metabolism is one of important carbohydrate metabolism pathway, which product propionic acid (Halarnkar and Blomquist, 1989), in the present study, higher propanoate metabolism compared with higher PA content in the first cutting silage.

The key enzymes plays important roles in various metabolic pathways. Fructokinase, Hexokinase, 1-phosphofructokinase, pyrucate kinase, lactate hehydrogenase are enzymes in glycolysis (Van Schaftingen, 2021), Under anaerobic conditions, pyruvate is reduced to lactate in a reaction catalyzed by lactate dehydrogenase (Blanco and Blanco, 2017). L-lactate dehydrogenase and D-lactate hehydrogenase in S_3 were higher but the LA were lower and BA were higher in S_3 which maybe induced by Acetyl-CoA. Acetyl-CoA was higher in S_3, it is known that Acetyl-CoA derived from glucose is the sole precursor for a variety compounds such as butyric acid, fatty acid, acetone, hexanoic acid (Zhu et al., 2022). In the metabolism of homofermentative LAB, glucose is metabolized to lactic acid *via* the Embden-Meyerhof pathway (EMP), and the heterofermentative LAB possesses the pentose phosphate pathway (PPP) (Abdel-Rahman et al., 2011). Glucose-6-phosphate dehydrogenase, phosphogluconate dehydrogenas, and L-ribulose-5-phosphate3-epimerase were mainly involved in the PPP pathway (Chesworth et al., 1998), it was speculated that the promotion of glucose-6-phosphate dehydrogenase and L-ribulose-5-phosphate3-epimerase of bacterial community in S_3 indicated that the higher relative abundance heterofermentative LAB in S_3.

## Conclusion

This study highlighted the variations in bacterial communities in F, W and ensiled alfalfa from three cuttings in 1 year in Inner Mongolia. Our results showed that, compared with F, wilting did not change but ensiling considerably decreased bacterial abundance and diversity. Bacterial community and fermentation quality were influenced by cutting times. Carbohydrate, amino acid metabolism and key enzymes of ensiled alfalfa bacterial communities were differ amnog three cuttings. Proteobacteria were more predominant in the third cutting, and the alterations in bacterial communities were driven by the DM of W, which potentially affected the pH, NH_3_-N, BA content and fermentation quality of S. The third cutting had the lowest fermentation quality. So, compared with the first and second cuttings, alfalfa from the third cutting was more likely to result in poorly preserved silage in central and western Inner Mongolia, China.

### Data availability statement

The datasets presented in this study can be found in online repositories. The names of the repository/repositories and accession number(s) can be found at: https://www.ncbi.nlm.nih.gov/, PRJNA827667.

## Author contributions

JS and ZY conceived and designed the research. JS, JW, CB, JZ, YY, YX, TZ, and WB conducted the experiment, JS, JW, and JZ analyzed the data. JS wrote the manuscript. All authors reviewed and edited the manuscript.

## Funding

This work was supported by Natural Science Foundation of Inner Mongolia (Grant no. 2022MS03011), Special Fund for Agro-Scientific Research in the Public Interest (201303061), Key Projects in Science and Technology of Inner Mongolia (Grant no. 2021ZD003), Agricultural Science and Technology Innovation Program of CAAS (No. 27-GRI-01), and Science and Technology Project from Inner Mongolia (Grant no. 2021GG0068).

## Conflict of interest

The authors declare that the research was conducted in the absence of any commercial or financial relationships that could be construed as a potential conflict of interest.

## Publisher’s note

All claims expressed in this article are solely those of the authors and do not necessarily represent those of their affiliated organizations, or those of the publisher, the editors and the reviewers. Any product that may be evaluated in this article, or claim that may be made by its manufacturer, is not guaranteed or endorsed by the publisher.
