# Supplementary material for: Natural fermentation quality, bacteria, and functional profiles of three cuttings of alfalfa silage in a year in Inner Mongolia, China
Source: Front Microbiol. 2023 Mar 9;14:1083620. doi: 10.3389/fmicb.2023.1083620 (PMC10033646; doi:10.3389/fmicb.2023.1083620)
Supplement: Supplementary file 1 [file Table_1.doc]

TABLE S1 Sequence and bacterial alpha diversity of fresh, wilted and ensiled alfalfa in three different cuttings.

|  | Observed OTUs | Shannon index | Simpson index | Chao 1 | Good’s coverage |
| --- | --- | --- | --- | --- | --- |
| F_1 | 156±5.03Ac | 3.74±0.68Aa | 0.76±0.12Aa | 221.28±21.57Ab | 0.997±0.0002Ba |
| W_1 | 148±14.41Ab | 3.93±0.11Aa | 0.84±0.01Aa | 187.17±14.42Ab | 0.997±0.0004Ba |
| S_1 | 67.66±12.66Bb | 2.91±0.44Aa | 0.71±0.09Aa | 86.69±15.53Bb | 0.999±0.0001Aa |
| F_2 | 249.33±6.43Ab | 4.28±0.41Aa | 0.85±0.04Aa | 311.91±5.62Aa | 0.995±0.0002Ab |
| W_2 | 274±21.14Aa | 4.74±0.37Aa | 0.88±0.04Aa | 337±22.24Aa | 0.995±0.0005Ab |
| S_2 | 139.67±16.61Ba | 3.28±0.48Aa | 0.76±0.07Aa | 187.25±14.68Ba | 0.996±0.0005Ab |
| F_3 | 302±6.12Aa | 5.25±0.20Aa | 0.94±0.005Aa | 345.80±1.70Aa | 0.995±0.0003Bb |
| W_3 | 264±42.96Aa | 4.39±0.38Aa | 0.85±0.03Aa | 306.34±46.68Aa | 0.996±0.0002Bab |
| S_3 | 148.67±12.21Ba | 3.64±0.91Aa | 0.74±0.12Aa | 183.48±21.11Ba | 0.998±0.0002Aab |

Good’s coverage: coverage is calculated as C=1-(s/n), where s is the number of unique OTUs and n is the number of individuals in the sample. This index gives a relative measure of how well the sample represents the larger environment. F, fresh alfalfa; W, wilted alfalfa; S, ensiled alfalfa; 1, the first cutting; 2, the second cutting; 3, the third cutting.
